# Living arrangement dynamics of older adults in Mexico: Latent class analysis in an accelerated longitudinal design

**DOI:** 10.4054/DemRes.2019.41.50

**Published:** 2019

**Authors:** Curtis Huffman, Ricardo Regules-García, Delfino Vargas-Chanes

**Affiliations:** 1 Development Studies Program, National Autonomous University of Mexico (UNAMPUED).; 2 (Population Council).; 3 Development Studies Program, National Autonomous University of Mexico (UNAMPUED).

## Abstract

**BACKGROUND:**

Because living arrangements have many implications for the well-being of older adults, knowledge regarding typical age-related developmental changes in living arrangements is of a major concern for public health policymakers, particularly in low- and middle-income countries dealing with growing aging populations. However, the much-needed empirical analysis of living arrangement dynamics is hindered by a lack of proper data.

**OBJECTIVE:**

To exploit often-available short-term longitudinal data in the study of long-term phenomena, in this paper we accelerate the Mexican Health and Aging Study (MHAS) panel as a means to explore, over a broad age span, the household dynamics of Mexican older adults.

**METHODS:**

Instead of working with a priori definitions of different household structures when analyzing transitions between them, we introduce a novel approach that estimates latent classes of developmental trends in the household composition of older people as they age.

**RESULTS:**

We show how accelerated longitudinal designs, coupled with latent class analysis, can offer new insights into living arrangement dynamics. Our findings suggest that in Mexico the typical living arrangements at 50 years old serve as an important predictor of future living arrangements, and that typical living-arrangement trajectories are strongly gendered in Mexico. This new approach may prove to be indispensible when determining the social support needed by high-risk population groups and as a means to better anticipate the necessary financial resources to do so.

## 1. Introduction

High-income economies have developed social security, pension, and public health systems to provide care and support to older adults. Conversely, in some low- and middle-income countries (LMIC) there is limited or no government-funded institutional support, and well-developed old-age support systems have yet to be created. In Mexico, older people frequently require social, economic, and physical assistance (DeGraff, Wong, and Orozco-Rocha 2018; Montes de Oca et al. 2014; Montes de Oca and Hebrero 2008), and many are unable to provide for themselves because of poor health and a lack of private savings, relying mostly on other family members for their well-being and survival (Ham Chande 2010, 2014; Nava Bolaños and Acosta 2018; Nava Bolaños, Ham Chande, and Ramírez López 2016).

The role of the family as a social institution responsible for the distribution of goods and services between generations has been widely theorized and discussed for a long time (Angel, Vega, and López-Ortega 2017; Becker 1991; Economic Commission for Latin America and the Caribbean (ECLAC) 2019; Kuznets 1978; Thornton, Chang, and Sun 1984). In many LIMCs (Bernabe-Ortiz et al. 2016), including Mexico (Lloyd-Sherlock et al. 2018), strongly gendered norms prevail regarding respect for older people and the responsibility of the young to care for the old as the need arises, Given these socially constructed and situational contexts, it is primarily older adults’ families that provide care and support.

Population aging has been recognized worldwide as one of the most significant demographic transformations of the 21^st^ century, and issues concerning care and support for older adults are of increasing importance. Aging in Mexico is no exception, particularly since there has been a remarkable decline in mortality, with life expectancy increasing from 33 years in 1920 (Parker, Saenz, and Wong 2018; Partida-Bush 2006) to 76 years in 2018 (González 2015). According to population projections from the Mexican National Population Council (CONAPO), the percentage of the population aged 65 and older is expected to grow from 6.7% in 2015 to 16.2% by 2050 (Regules García 2018). This demographic trend will increase cost pressures associated with aging, such as medical and long-term care expenditures.

Providing universal health insurance coverage is a major challenge in most LMICs, given that the informal sector absorbs the great bulk of the labor force (Bitran 2014; Parker et al. 2018). In Mexico, social security provides health benefits for only a fraction of the population (Parker, Saenz, and Wong 2018), and hence older people without social security rely mainly on public transfers from the Ministry of Health or familial transfers.

Mexico’s inward-looking development strategy produced sustained economic growth from the 1940s until the late 1970s. However, since the early 1980s the country has been experiencing significant recessionary setbacks that have affected the dynamics of coresidence. Coresidence of adult children, parents, and other family members has been identified as an economic survival strategy (Cortés 2000; Cortés and Rubalcava 1991; Chant 2007; González de la Rocha 1994; Singh 2002). In a context of widening socioeconomic disparities in healthy aging and longevity (Bobadilla et al. 1993) combined with low old-age and retirement pension coverage – about 30% of those aged 65 and older (Ramírez López, Nava Bolaños, and Badillo González 2018) – the reliance of older people on old-age security from family and kinship networks, particularly adult children, will likely increase.

Family life-cycle events, such as changes in marital status (divorce/separation, widowhood, remarriage), migration, gender differences in mortality, and precarious socioeconomic conditions may result in aging parents and adult children still living at home, grandparents raising grandchildren, and a growing trend of older women living alone. These living arrangements are associated with the health and well-being of aging populations (Gruijters 2017; Manfredini and Breschi 2013; Montes de Oca et al. 2014; Montes de Oca and Hebrero 2008; Teerawichitchainan, Pothisiri, and Long 2015; Tohme et al. 2011). An understanding of the living arrangement dynamics of older adults is urgently needed in order to design public policy on aging and to improve multi-faceted interventions aimed at improving quality of life in old age. However, the empirical analysis of the dynamic phenomena associated with household life cycles requires access to information that follows individuals and households over a long period of time – information that is of limited availability, especially in LMICs.

In Mexico, the Mexican Health and Aging Study (MHAS) provides the opportunity to follow households and individuals over a period of up to 15 years (Wong, Michaels-Obregon, and Palloni 2015). Between 2001 and 2015 households were interviewed up to four times at irregular time intervals. In fact, for most households in the MHAS the observed period is far shorter than 15 years, giving not much more than a snapshot of the dynamics surrounding their living arrangements. As a result, there has been limited use of the panel aspect of the MHAS when analyzing household dynamics (Montes de Oca and Hebrero 2008).

Another factor behind the limited attention that has been paid to panel analysis is that when studying living arrangement dynamics, the information regarding changes in household composition gives rise to a large number of different trajectories that cannot straightforwardly and non-arbitrarily be captured in a manageable number of categories, even with a small number of measurement occasions.

In this paper we overcome the limitations of the survey’s time frame and the large number of different living arrangement transitions observed. We propose and illustrate methods that can extract understandable data patterns for further use in longitudinal studies. In a nutshell, first we accelerate the MHAS by rearranging it according to a cohort-sequential design, a technique that allows linking adjacent segments of limited longitudinal data from different age cohorts (Duncan, Duncan, and Strycker 2013). We then use Latent Class Analysis to estimate common developmental trends in the household composition of older adults as they age. Thus, our approach allows us to explore typical living arrangements trends among the Mexican elderly in a dynamic setting over a 30-year window with a simple cursory look. As shown below, our statistical framework also allows researchers to control for cohort effects, which is a major advantage over other approaches.

The results underscore, among other things, the stability of each living arrangement history – something that has not been done before. Although this may seem surprising at first, it is important to note that it does not necessarily imply that individuals rarely experience abrupt changes in the composition of their household, but that these changes are rarely correlated with age. Our results also suggest that public policies may be more effective if they are targeted at particular individuals. For example, if they are targeting most-at-risk populations (physically or financially) the at-risk population may be different when looking at household structure, which may change over time.

The rest of the paper is organized as follows. Section 2 presents and discusses current information on the living arrangements of older people in Mexico and Section 3 situates our methodological contribution within the study of the household dynamics and living arrangements of older adults. Section 4 describes the data and methods and describes the accelerating procedure and the proposed Multigroup Latent Class Analysis. The results are discussed in Section 5, and Section 6 presents our conclusions.

## 2. Background: Living arrangements of older adults in Mexico

In Mexico the population aged 65 and over is one of the most rapidly growing in the world. In 2018, older adults comprised roughly 8.3% of the total population; that is, 10.4 million older adults. One in five households surveyed reported having at least one older adult (65+). Projected estimates show that the total Mexican population will reach 150 million in 2050 while the group 65+ will reach 28.7 million (Angel, Vega, and López-Ortega 2017; Ceballos Mina 2017; Nava Bolaños, Ham Chande, and Ramírez López 2016; Payne 2015; Sánchez and Escoto 2017). Such a trend, if sustained, will certainly lead to different survival strategies, including multigenerational living arrangements (Ceballos Mina 2017; Redondo, Garay, and Montes de Oca 2015; Sánchez and Escoto 2017). In a recent study on household composition and aging in Mexico, Montes de Oca et al. (2014) find that nuclear (36.4%) and extended (46.3%) households are the most common households among older adults aged 60 and over. However, the authors note the presence of a significant proportion of single-person households (16%). This has been a stable trend since the 1990s, according to the National Survey of Demographic Dynamics (ENADID) (Redondo et al. 2015) and the MHAS (Montes de Oca and Hebrero 2006, 2008).

Little research has been done on changes in the living arrangements of the Mexican elderly, and even less has looked at these changes as a function of age. A couple of exceptions are the works of Montes de Oca and Hebrero (2006) and Ybáñez Zepeda, Vargas Valle, and Torres Martín (2005). The first uses longitudinal data from the Mexican Health and Aging Study (MHAS) to look at individuals in their 60s, 70s, and 80 years and older, and finds a significant decline in the proportion of nuclear households. This might be thought an almost natural change as children grow older and leave the parental home. The second study shows a similar trend with small differences between rural and urban areas (Ybáñez Zepeda, Vargas Valle, and Torres Martín 2005). It is noteworthy that in spite of the obvious potential links, very few researchers have taken on the challenge of analyzing the systematic tendency of the Mexican elderly to change living arrangements as a function of age (Pérez Amador and Brenes 2006).

As mentioned in the previous section, in Mexico many older adults are unable to provide for themselves because of a lack of private savings and limited or no access to a retirement pension, forcing them to rely mostly on other family members (Ham Chande 2010, 2014; Nava Bolaños and Acosta 2018; Nava Bolaños, Ham Chande, and Ramírez López 2016). In 2014 only 1 in 4 older adults aged 65 and over had a retirement pension, and only a small fraction of those were women (Angel, Vega, and López-Ortega 2017; Nava Bolaños, Ham Chande, and Ramírez López 2016). In Mexico the social protection system is tied to the formal labor market, and because the informal labor market is extensive this leaves a large segment of Mexican elders without access to a safety net and reliant on social and public health programs. According to Nava Bolaños, Ham Chande, and Ramírez López (2016), approximately half of the population aged 65 and over receives transfers from the government through social programs and, based on official poverty measurements, some 45.9% are living in poverty. Thus, older adults’ income and access to healthcare services is heavily dependent on scarce and unreliable social programs and networks (Aguila et al. 2011; Garay Villegas, Montes de Oca Zavala, and Guillén 2014; Salgado-de Snyder and Wong 2007).^4^

In the particular context of underdeveloped social security systems, which is not unique to Mexico, identifying and understanding the living arrangement dynamics of older people becomes extremely important for both low- and middle-income countries (Lloyd-Sherlock et al. 2018). According to Ullmann, Maldonado Valera, and Nieves Rico (2014), in such dissimilar countries as Uruguay, Bolivia, and Mexico, multigenerational households (those with individuals both under 15 and over 65 years old) exhibit the greatest economic vulnerability. As noted above, in Mexico this kind of living arrangement has been identified as an economic survival strategy in several studies and across different data sets (Garay Villegas and Montes de Oca Zavala 2011; Montes de Oca et al. 2014; Nava Bolaños, Ham Chande, and Ramírez López 2016; Sánchez and Escoto 2017; Ybáñez Zepeda, Vargas Valle, and Torres Martín 2005). Multigenerational households may increase social support through stronger structural ties in which resources (monetary, care, food, etc.) may be shared among many people, including older adults. This can reduce psychological stress by both conferring support and reducing financial strain (Kawachi, Subramanian, and Kim 2008). It is thus only natural that multigenerational arrangements are most notable among the most vulnerable populations ‒ those with less financial, social, or intellectual capital (Muennig, Jiao, and Singer 2018). Indeed, there is little doubt that informal labor trajectories, destitution, and poor health have a high correlation with living arrangements, but also that we know little about the dynamics behind it as a function of age.

## 3. Empirical approaches to the living arrangements of older adults

A survey of the literature on older adults’ living arrangements suggests that most of the studies implement a basic comparative scheme of mutually exclusive categories, usually breaking households down according to kin relationships and cohabitation based on a couple of demographic markers typically found in household surveys. However, even with only a few demographic markers, there are several quite distinct ways to operationalize the typology of living arrangements (De Vos 1990; Montes de Oca et al. 2014; Montes de Oca and Hebrero 2008; Montes de Oca Zavala and Nava Bolaños 2017; United Nations Department of Economic and Social Affairs/Population Division 2005; Varley and Blasco 2003).

Without a doubt, the choice of living arrangement typologies (marital status, family type, household type) depends on various theoretical and practical factors, including data availability and model complexity. However, whichever typology is chosen, once a decision has been made a key question remains: to what extent does the preferred typology reflect the actual household structures of older people? Unfortunately, even with a small number of demographic markers with just a few levels each, eyeballing the answer to this question from the *n*-way contingency table can be a formidable task, as the number of combinations grows exponentially with every demographic marker included. Seeing it as a dynamic process only adds further complexity. Perhaps this is why most studies on living arrangements rely on cross-sectional designs.

There are exceptions in the literature on older people’s living arrangements that which use longitudinal data to analyze family life-cycle transitions and changes in family size (Brown et al. 2002; Dostie and Léger 2005; Keilman and Prinz 1995; Martikainen, Nihtilä, and Moustgaard 2008). However, these studies also rely on a-priori-defined types of family and household structure. It is important to note that in these dynamic models every judgment regarding the relative stability of older people’s living arrangements is predicated on the chosen typology. In practice the choice usually made is a reasonable compromise between the conflicting objectives of completeness (the complexity required to actually track the observed changes) and feasibility (the parsimony required, for a given sample size, to fit the probability models).

In the analysis that follows, as a way to circumvent the restrictions imposed by a priori postulated types of household structure, rather than using a priori household structure definitions it is possible to identify empirically the main household types and their typical sequences through a Latent Class Analysis (LCA) (see Wang and Wang 2012 and Hagenaars and McCutcheon 2002). This technique profits from the correlation between observed household characteristics, both cross-sectional and longitudinal, and allows identifying household structures by empirically seeking the best way to represent the observed household demographic structure. An advantage of LCA is that it broadens the set of demographic variables as needed in order to identify household structure by including continuous, ordinal counts or categorical variables (Oberski 2016).

Du and Kamakura (2006) follow this path, identifying distinct household structures through an LCA with a Hidden Markov Model (HMM). Although the HMM paves the way for a better understanding of the relationship between age and living arrangement transitions that occur over time, it has important limitations when describing the main developmental trends commonly observed in the living arrangements of older adults. First, in contrasting the living arrangement at time *t* + 1 to that at time *t*, it forgoes the opportunity to look at a longer (or finer) set of consecutive events if using longitudinal surveys with two or more waves. Thus, the HMM does not provide enough information on the transition process itself. For example, HMM will not tell us about how long it takes respondents to experience living arrangement transitions or how this process varies with age. This poses a problem, because the changes being modeled are not necessarily an accurate representation of the developmental patterns/trajectories in household compositions as individuals age, but rather those from measurement occasion to measurement occasion – wave to wave in the survey. Second, when pooling the data from different measurement occasions, the HMM makes it very difficult to control for cohort effects.

Unlike previous studies, this paper attempts to fill this void by presenting results from an exploratory analysis of the living arrangement dynamics of older people in Mexico based on an Accelerated Longitudinal Design (ALD). This novel approach expands our current knowledge concerning the living arrangements of older adults, allowing us to start unpacking the dynamic processes through which individuals arrive at a particular household structure as they grow old.

In order to provide more detailed information about the living arrangement transition process and how that process varies over the latter part of the life course, we follow Du and Kamakura (2006) and examine living arrangements from a dynamic perspective by means of LCA. Thus, we also use Structural Equation Modeling (SEM) to classify households into different categories/types according to a combination of variables usually considered to be indicators of particular household structures. In this sense we also estimate, rather than postulate, the typical dynamics behind the household structures observed in the country.

However, unlike Du and Kamakura, we do not estimate an HMM. Instead, we use LCA to estimate the typical conjoint developmental trends of demographic markers, which allows us to recognize the main types of living arrangement dynamic. In this way we make full use of the longitudinal design of our data, exploiting the correlation of our sociodemographic variables through time – not just between time *t* and *t* + 1, but through all measurement occasions as a function of age – while controlling for cohort effects. This provides us with a more complete analysis of the living arrangement dynamics of the population aged 50 and over.

## 4. Data, measures, and methods

For our analysis we use data collected by the Mexican Health and Aging Study (MHAS). The MHAS gathers detailed longitudinal information about the aging process of people aged 50 and over. The study also collects sociodemographic data for all spouses (regardless of age) and household and family members, and life histories for each age-eligible (≥ 50 years old) person. The first wave of the survey was conducted in 2001, and follow-up waves were conducted in 2003, 2012, and 2015. Our analysis is based on 18,845 individuals aged 50 and over.

The structure of households has traditionally been analyzed by the relationship of individuals to the head of household or in terms of their size. However, often the household does not have a well-defined starting or finishing point for longitudinal analyses (Murphy 1991). In this article we adopt an unconventional approach to analyze the living arrangement dynamics of older people. First, we examine the relationship of individuals to the age-eligible (≥ 50 years old) person. Second, we perform a Latent Class Analysis (LCA) based on an Accelerated Longitudinal Design (ALD) to analyze the typical living arrangement trajectories as the older adult population ages.

### 4.1 Accelerated longitudinal design (ALD)

Longitudinal survey designs have many advantages over cross-sectional designs, most notably in providing information about within-individual change (Duncan, Duncan, and Strycker 2013). However, longitudinal surveys also pose major potential problems, such as confounding aging and period effects, delayed results, not achieving continuity in funding and research direction, cumulative attrition (Farrington 1991), the amount of time required to collect enough data about each individual to analyze social and demographic phenomena, and particularly the time span required to study changes in family and household structures.

**Figure A-1:**
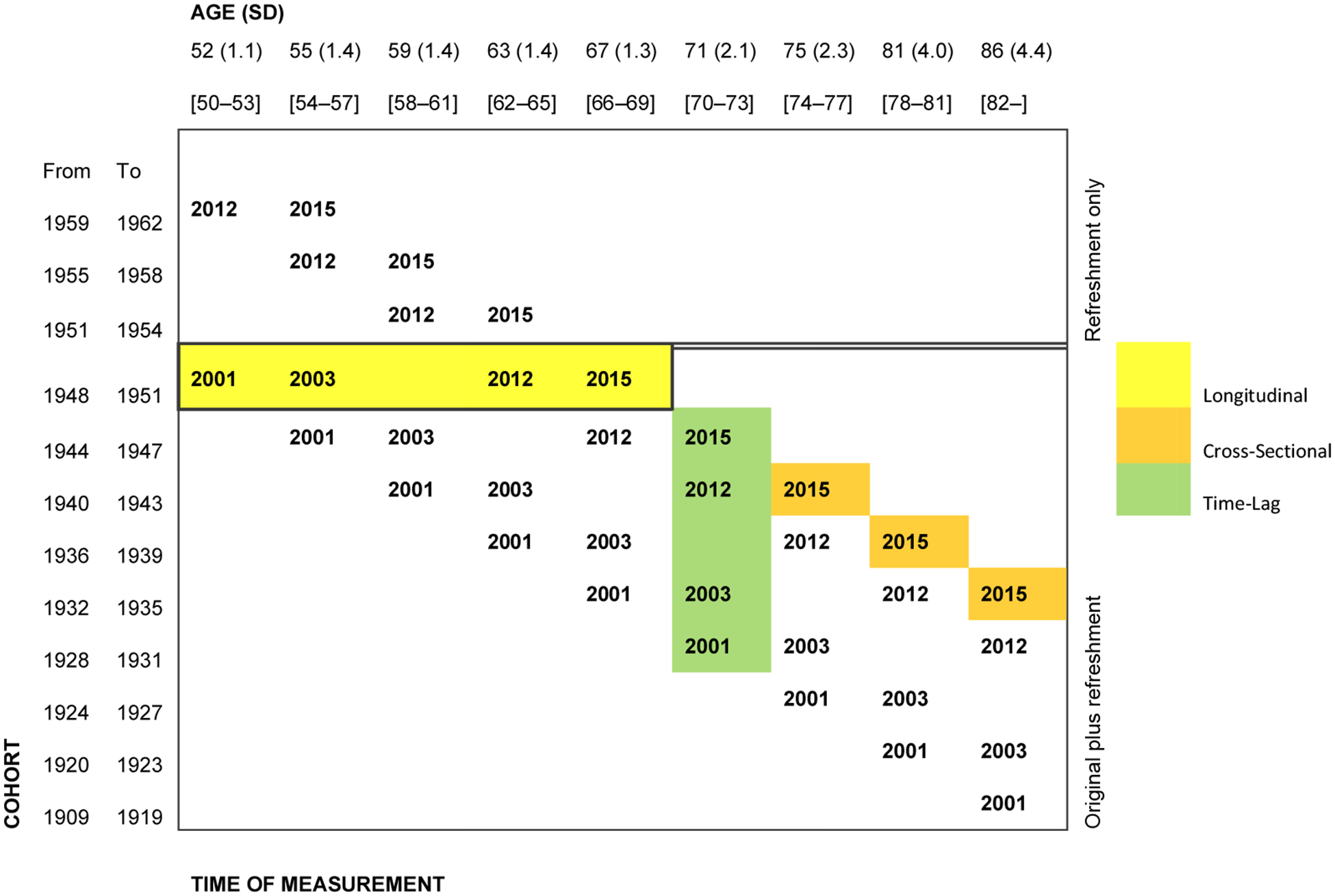
Illustration of MHAS-ALD

Bell (1953)^5^ first proposed the strategy referred to as Accelerated Longitudinal Design (ALD) as a means to overcome longitudinal survey problems. Bell’s proposal consists of collecting limited repeated measurements of independent age cohorts and temporally overlapping the cohort data (see Appendix Figure A-1). In this way researchers can approximate a long-term longitudinal study by conducting numerous concurrent short-term longitudinal studies of different age cohorts (Duncan, Duncan, and Hops 1996). An obvious advantage of the ALD is that it has the benefits of the longitudinal methods while minimizing time, continuity, attrition, and budget constraints.

In this article we explore, for the first time, the potential benefits of using an ALD to study the living arrangement dynamics of older adults. The MHAS baseline survey was conducted in 2001, and follow-up interviews were conducted in 2003, 2012, and 2015. In 2012, panel data sets were supplemented by an additional refreshment sample to deal with attrition due to death, decline in the health of participants, and study dropout across the follow-up waves. To minimize the number of time periods (segments of the life span) under analysis while keeping track of the different waves of the study, we divide the index older adult population into nine age groups. Table 1 shows the number of observations by wave and respondent’s age group.

Because overlapping age across measurement occasions occurs, Table 2 displays intersecting cohorts.^6^ Cohort 1 [1909‒1919], ages 82‒92;^7^ Cohort 2 [1920‒1923], ages 78‒107; Cohort 3 [1924‒1927], ages 74‒81; Cohort 4 [1928‒1931], ages 70‒113; Cohort 5 [1932‒1935], ages 66‒91; Cohort 6 [1936‒1939], ages 62‒81; Cohort 7 [1940‒1943], ages 58‒77; Cohort 8 [1944‒1947], ages 54‒73; Cohort 9 [1948‒1951], ages 50‒69; Cohort 10 [1951‒1954], ages 58‒65; Cohort 11 [1955‒1958], ages 54‒61; and Cohort 12 [1959‒1962], ages 50‒57.

The mean overlap between consecutive waves is 57%, meaning that more than half of the observations reappear in the first follow-up survey conducted in 2003, half of them in a second survey in 2012, and one-third in the last follow-up survey in 2015, that is, 15 years after the baseline survey (2001) was completed. Our analytical sample includes 18,845 participants scattered throughout 12 cohorts. The cohort sample sizes range from n = 11 for Cohort 1 to n = 10,220 for Cohort 9 (see Table 2). Overall, 46% of the sample were men and almost half of them (48%) were between 51 and 58 years old when responding to the baseline survey in 2001.

Table 3 presents changes across time-period (index older adult mean age) in descriptive statistics for selected household demographic variables using data from the ALD.

Regardless of overlapping cohorts and relatively large sample sizes, it is important to note that each cohort displays a unique pattern of missingness (see Table 2). In this context, the question immediately arises of whether cohorts are sufficiently comparable to provide reliable information to characterize the living arrangement dynamics of older people between 50 and 90 years of age in Mexico,^8^ and if the ALD allows us to differentiate between age and cohort effects. Regarding the latter, an advantage of the ALD is that since different cohorts of the same age in different periods can be linked together to represent a longitudinal trend, aging may be analyzed independently of period and cohort effects.^9^

Regarding comparability, subjects in the same overlapping time span that belong to different cohorts should be as similar as possible in order to strengthen confidence in our estimated longitudinal trends (Duncan, Duncan, and Hops 1996). If the cross-sectional measures of the ALD exhibit different patterns across cohorts, they can lead to misleading conclusions about aging effects on the living arrangements of older adults.^10^

The fact that our analytical approach uses several demographic household variables poses a reasonable doubt regarding the naïve assumption that cohort effects are null or small enough to be dismissed. Thus, in this article we assume that age and cohort interact, confounding the living arrangement dynamics of older people, and therefore control for these effects in our models.

In particular, inferences regarding correlates of change are adjusted for cohort membership, exploiting the flexibility of structural equation modeling (SEM) to view cohorts as subpopulations. In the next section we address the validity of our methodology to characterize the evolution of household dynamics (living arrangements).

### 4.2 Latent class analysis (LCA)

Our approach to estimating the typical trajectories of Mexican older adults’ household structures involves investigating the MHAS-ALD for the presence of subgroups, or classes associated with different patterns of demographic markers as individuals age.

To characterize household structure trajectories, apart from the sex of the respondent – still a major factor in the possible living arrangements of older people in Mexico – we used nine household-level sociodemographic markers to profile the dynamics of the respondents’ living arrangements in terms of cohabitation, kin, and economic-dependence relationships within the household.

For each time point (age bracket of the respondent) in the ALD we use the following variables: (1) Children living in the household; (2) Children ≥ 18 years old;(3) Studying children ≥ 18 years old; (4) Working children ≥ 18 years old; (5) Children’s partners; (6) Index older adult’s partner or spouse; (7) Nonrelative; (8) Other relatives; and (9) Grandchildren and/or great-grandchildren.

Latent Class Analysis (LCA) offers a valuable approach to the task at hand. LCA allows us to identify classes (typologies) of household structure trajectories from the relationships between this set of sociodemographic variables. Loosely speaking, LCA partitions the original ALD into subsets with like developmental trends in their sociodemographic markers. This has the added value of further exploiting the utility of the ALD, as it links segments consisting of limited longitudinal data on a specific age cohort with similar segments from other temporally related age cohorts in determining classes of common household structure developmental trends – much as a matching procedure would. In other words, LCA groups observations in line with what one would expect from an ALD that provides an accurate picture of age-related change.

In this sense, LCA helps deflate the importance of Age X Cohort interaction effects within classes, thus hampering potential cohort differences in developmental trajectories. However, given the different missing patterns of individuals in an ALD, as cohorts differ from one another, LCA may end up classifying the bulk of one cohort in a single class, with marginal contributions to the others. The downside of this eventuality is the entanglement of age effects with cohort effects.

To circumvent the issue of confounding age with cohort effects, rather than examining a posteriori potential cohort differences in the estimated developmental trajectories – Miyazaki and Raudenbush (2000) refer to this type of examination as the “test of convergence” – we imposed several restrictions in a multiple-group^11^ approach to impose measurement invariance and equal transition probabilities across cohorts, thus controlling for age–cohort effects directly in our model structure.

In other words, by viewing cohorts as subpopulations, we adapted the multiple-group approach in order to estimate classes of household structure trajectories by imposing a common classification scheme. That is, we restricted the model to be fitted so as to make the probability of belonging to a particular class the same across cohorts, thus making it impossible to predict class from cohort.

### 4.3 Multi-group Approach to LCA

Standard LCA models are characterized by two parameters: the probability of a randomly selected individual in the population being in a particular latent class, and the probability of a member of a particular latent class (LC) yielding a particular set of responses to the observed variables. As an extension of this traditional approach, multi-group LC models assume the presence of three types of categorical variables: observed (indicator) variables, in our case the household demographic markers (***M***) at each of the 9 time points (age bracket of the individual) with two levels (*l* = 1,2); an unobserved (latent) variable that accounts for the relationships between the observed variables, this being the class of household structure development *X* with S classes (*s* = 1,2, … ,*S*); and a grouping variable *G*, in our case the cohort to which individuals belong, which categorically scored, manifest variable, indexed by *c* = 1,.. ,*C*, which can be associated with both the demographic markers *M*_*it*_ and the latent class to which it belongs. The 10 demographic markers ***M*** are observed indexed by *i* = 1, .. ,10 and by time point (*t* = 1, … ,9) with different patterns of missing values in each cohort *G*. Note that even though in principle we have a set of 13 90-way (*M*_1,1_ × … × *M*_10,1_ × *M*_1,2_ × … × *M*_10,2_ × … … × *M*_1,9_ × … × *M*_10,9_) observed contingency tables, no single observation has data on all ***M***_*t*_, leaving a lot of empty cells in the contingency tables. The multigroup LC model is presented in Equation 1:

(1) $$\pi_{lsc}^{MX|G}=\pi_{sc}^{X|G}\prod_{i}\prod_{t}\pi_{lsc}^{M_{it}|XG}.$$

Here $\pi_{lsc}^{MX|G}$ denotes the conditional probability that an individual who belongs to cohort *c* will be at levels (*l*_1,1_, … *l*_10,9_, *s*) with respect to variable ***M*** and *X*. The conditional probability of *X* taking on the level *s* for a member of cohort *c* is denoted by $\pi_{sc}^{X|G}$. The proportion of LC for the cohort *c*. $\pi_{lsc}^{M_{it}|XG}$ is determined by the conditional probability of an individual taking the level *l* in demographic marker *M*_*it*_ for a given class and cohort membership. Note that in the model presented above all the parameters differ across cohorts, making it difficult to compare the results across groups.

In order to make the data coming from different cohorts as comparable as possible, we have restricted equal class-specific conditional response probabilities across cohorts. That is, we have imposed equality restrictions on these conditional probabilities, thus imposing a homogeneous measurement structure across cohorts.

Specifically, all parameters are restricted so that they are equal across cohorts; that is, the conditional probability of items $\pi_{ls1}^{M_{it}X|G}=\pi_{ls2}^{M_{it}X|G}=\cdots =\pi_{lsC}^{M_{it}X|G}$, making the indicator variables independent of the cohort variable when controlling for the latent variable, and the conditional probability of the latent variable $\pi_{s1}^{X|G}=\pi_{s2}^{X|G}=\cdots =\pi_{sC}^{X|G}$.

In other words, we have estimated the case of a completely equivalent model.^12^ Note that by explicitly imposing these restrictions on the analytical model, cohort differences in age-related change in household composition trajectories would, in principle, be null. This is not the same as disregarding the cohort to which each observation belongs in our models. On the contrary, we have imposed a homogenous model on every cohort, in a probability model in which it is impossible to predict class from cohort, thus avoiding the case in which a latent class comprises the bulk of a particular cohort.^13^

Even with these restrictions, the parametric model can be computationally intense and take a nontrivial amount of time to run, because a threshold parameter must be estimated for each of the 90 indicator variables. In some instances this model complexity can lead to nonconvergence problems or improper solutions.

To ease the computational burden and convergence of our LCA models, in order to balance the number of observations across cohorts and reduce the number of parameters, data for the MHAS-ALD was aggregated in three different groups according to decade of birth: G1, cohorts 1‒6, basically all respondents born before 1940; G2, cohorts 7‒9, roughly all respondents born in the 1940s; and G3, cohorts 10‒12, born in the 1950s.

## 5. Results

Since the number of latent classes of living arrangement trajectories is unobserved, it cannot be estimated directly from the MHAS-ALD. Thus, the first step in our analysis is to determine the optimal number of trajectory classes to analyze. To determine the optimal number of latent trajectories we followed the common practice of fitting a series of LCA models with an increasing number of latent classes, comparing the *k*-class model with the (*k* − 1)-class model iteratively.

Following Lo, Mendell, and Rubin (2001) and Cortés and Vargas-Chanes (2016: 62‒63), we used two model fit indices and statistics to determine the number of trajectories to analyze, as shown in Table 4: Log-likelihood (2) and Bayes Information Criterion (3) (Schwarz 1978). We did not use the Lo-Mendell-Rubin likelihood ratio, given the complexities of its interpretation under the multigroup approach.

We also examined the quality of the latent class membership classification in each of the seven models looking at: a relative entropy criterion (4) (Wedel and Kamakura 2000), which is a rescaled version of Celeux and Soromenho (1996); the relative class size or the percentage of individuals in each class (5); and the range of the estimated individual probabilities of belonging to a specific latent class (6). In addition, when making choices we considered the theoretical meaning and interpretability of the classes estimated in each model in light of the country’s demographics.

We specified up to 5 latent classes.^14^ Table 4 reports model fit indices and statistics, which suggest that the model with 4 classes is a good candidate, given that the smaller values of information criterion indices (BIC) indicate a better model fit than for that with 3 classes and it has higher entropy and a higher classification rate than that achieved with a 5-class model.

Even though the 3-class model also seems a good candidate for analysis, we will discuss the results of the 4-class model, since the extra class allows us to look closely at the trends followed by larger households (Class 1 below) separately. As already noted, when selecting the number of classes it is important to consider not only the statistical indicators but also the substantive meaning of each class, including the nature and ultimate objective of subsequent analyses.

**Figure 1:**
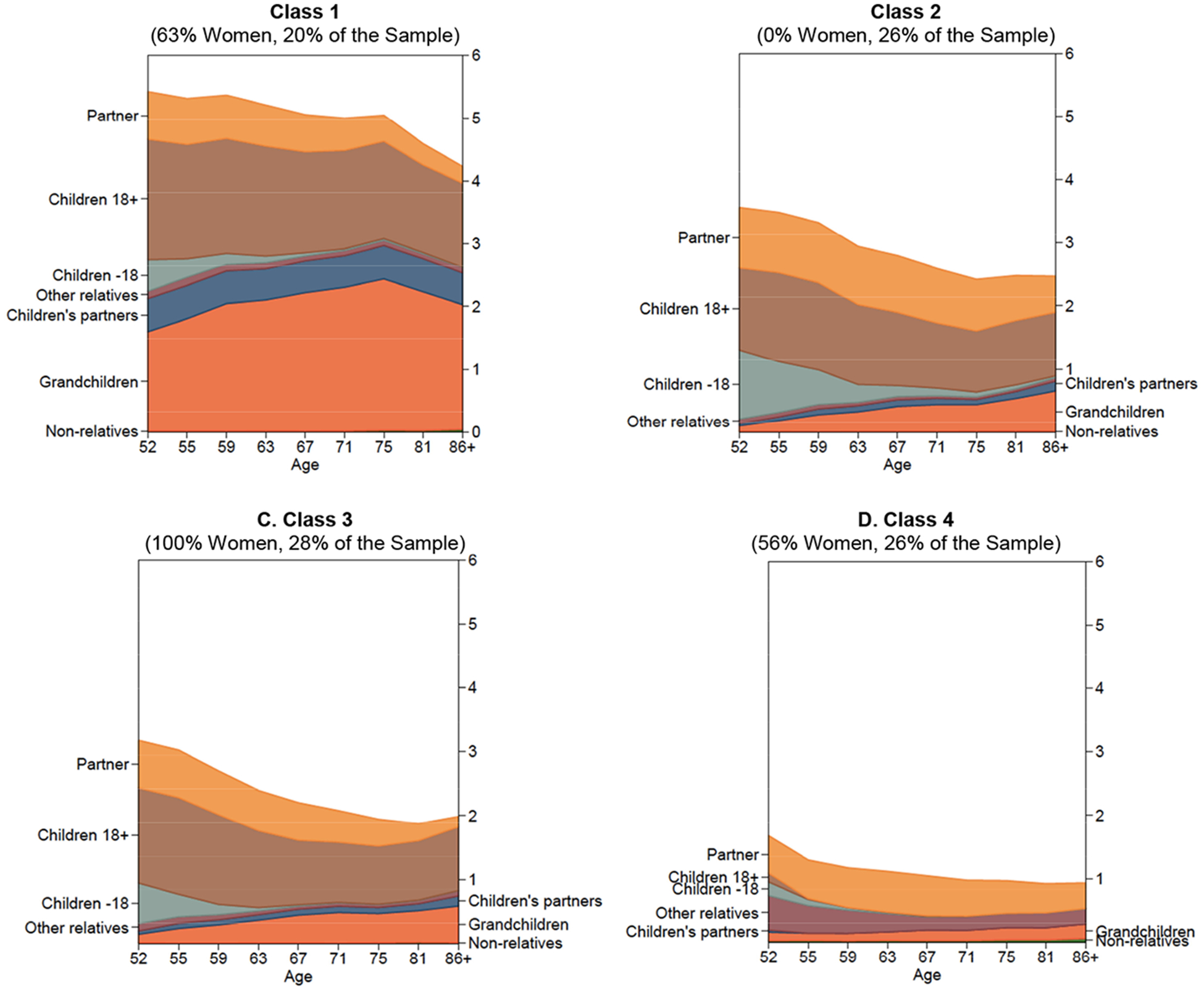
Estimated living arrangement trajectories *Source*: Prepared by authors based on data from MHAS I-IV.

Figure 1 depicts the different developmental trends of Mexican older adults’ household composition according to Tables 6–9 in Appendix B. There we can see the average composition of the respondent’s household as they grow older – but not the respondents themselves.

The different panels in Figure 1 present the individual’s age along the x-axis and the average number of other household members along the y-axis, according to their familial relation with the respondent: the number of children, older adult’s partners in the household, children, children’s partners or spouses, grandchildren, other relatives and nonrelatives.

Looking at these results we can see that Class 1 trajectories include older adults, slightly more women than men, and living in the largest households with an average size of 6 persons; however, the average household size fell from 6.4 at age 52 to 5.2 at ages around 86 (see Figure 1, panel A). Here it is important to note that even though we made no effort to control for locality size, all classes end up with a distribution that closely follows the national average. In other words, it is not the case that a relatively larger number of rural households are concentrated in Class 1 (see Table 5 in Appendix B).

Compared with Classes 2, 3, and 4, it is noteworthy that the average household size in Class 1 declines sharply only after 75 years old, whereas among the other Classes a noticeable decline in household size begins much earlier, around 52 years for Class 4 or after 60 years in Classes 2 and 3. Note that Class 1 has the highest average household size, while the size of the average household declines from 4.6 to 2.7 as we move from Class 2 to Class 4.

Households in Class 1 register the highest number of grownup children (aged 18 and over), as well as the highest numbers of children’s partners/spouses and grandchildren. Eight out of 10 households in Class 1 have three generations living together, and this does not change as the respondents grow older, even though these households tend to get smaller as the respondent turns 75.

While it is true that Classes 2 and 3 also register high numbers of grownup children, unlike those in Class 1 they start mostly single and with hardly any children of their own living with them, although by age 86 we can find a children’s partner in 15% of these households and grandchildren in a quarter of them. It is noteworthy that, contrary to Class 1, the presence of grandchildren in these household increases after the respondents turn 75 years old.

Class 2, comprised mainly of men, exhibits the highest frequency of children below the age of 18, mostly young adolescents. Unlike in any of the other classes, it is still possible to find children below the age of 18 in 1 out of 10 households by the time respondents turn 67. In turn, households in Class 3, comprised mainly of women, are slightly smaller and children under 15 are harder to find. The fact that in these classes the average number of children under the age of 18 living in the household falls more rapidly than the number of grownup children rises speaks to us of the pace at which children, as they grow up, leave the household, go to college, or form their own households.

In all four classes we observe common trends in household size, the percentage of households where the older adult’s spouse is present, and the presence of children under the age of 18: all decline as respondents grow older. Cohabitation is most notable for women in Class 3. It is important to note that changes in the presence of a partner in the household could mean a combination of either the death of the partner/spouse or, in some cases, divorce or separation, and the formation of a new partnership. In this sense, the peculiarity of Class 3 may be partially due to the characteristic supermortality of women (women often outlive their spouses) in combination with a higher reluctance to repartner.

In addition, Class 4 stands out as having the smallest average household size but the highest number of other relatives living in the household, growing steadily in proportion from 1.1% to 3.8%. The combination of a growing presence of grandchildren and a decreasing number of children in the household reminds us that migration to the US is an age-specific phenomenon in many areas of Mexico that may disrupt traditional living arrangements and increase the likelihood that individuals live alone and/or with their grandchildren and other relatives.

Also important to note is that in all four trajectories the percentage of households with grandchildren increases as respondents grow older, and that in ¾ of our sample (except in Class 4) it is possible to find Mexican older adults living with at least one of their children throughout the whole period. This might reflect high levels of family caregiving for the older adult and a high degree of continuity in parent–child coresidence trends, so that a parent is likely to live with one or more children (Kanaiaupuni 2000), their children’s partner or spouse, and their grandchildren. Although this pattern appears consistent in 3 of the 4 classes, it is most visible in Class 1, which has the largest average household size.

The results are relevant in terms of public and social policy because parent–child coresidence in Mexico is common, due both to structural constraints fed by economic instability and limited services and institutional coverage for the poor and older people, and to social norms that emphasize the importance of the family (Kanaiaupuni 2000; Varley and Blasco 2003).

At a minimum, these results provide us with additional information to feed the discussion of the dynamics of Mexican older adults’ living arrangements. Depending on the ultimate research questions researchers might want to address, in combination with goodness-of-fit indices, it is possible to look into other models, with a different number of classes, to better inform public policy.^15^

## 6. Conclusions

As living arrangements have many implications for the well-being of older people, knowledge regarding their typical age-related developmental changes is of major concern for public health policymakers, particularly in societies dealing with growing aging populations and relatively underdeveloped social security systems.

This paper approaches the study of older adults’ household dynamics and living arrangements in Mexico in a novel way on several fronts, both methodological and empirical. First, we have shown how it is possible to further exploit the Mexican Health and Aging Study (MHAS), and surveys like it, by using an Accelerated Longitudinal Design to look at household structures as a crucial determinant of older adults’ well-being. Second, we have shown how by falling back on Latent Class Analysis (LCA) it is possible to overcome some of the methodological limitations previously encountered in the empirical literature on household dynamics. Third, we have proposed a way to control for cohort effects in an ALD framework by means of a multigroup approach to LCA. Last, we have contributed to the empirical literature on older people’s household dynamics in Mexico by estimating typical developmental trajectories of their living arrangements in a 30-year time span; that is, for the first time we have estimated typical full living arrangement histories of Mexican older adults.

These methodological innovations allow researchers to answer questions concerning typical living arrangement trajectories that have not been addressed hitherto. More specifically, the ALD-LCA approach allows researchers to address questions such as: How likely is it that an older adult in a particular living arrangement and at a certain point in his/her life is in a particular living arrangement trajectory? How are living arrangement’s developmental trajectories related to age and distributed over the latter part of the life course? Similarly, at what pace do these living arrangements change? How long can an individual expect to live in a particular living arrangement before experiencing a significant change?

It is important to note that here we have used the ALD-LCA approach in a very descriptive way, looking for typical developmental trends in living arrangements, whereas the probability model estimated within this framework easily dovetails with other causal inference and decomposition techniques. Identifying typical living arrangement trajectories may prove a first step in putting on a better footing our causal estimates of the effect of household structure on the elderly’s well-being. A byproduct of the approach we have taken here is a probability model that can be exploited in a propensity score framework (Guo and Fraser 2015). This allows for more elaborate models where the search for typical trajectories can be made (conditioned) to directly answer specific questions regarding particular outcomes, such as which living arrangement’s developmental trajectory relates to a particular outcome growth curve. As long as sample size and data set variability allow it, the analytical possibilities are many.

Demographic changes resulting in the aging of the population, along with changes in demographic behaviors such as migration, marriage, and childbearing, have transformed the intergenerational structure of society and contributed to changes in living arrangements. This in turn raises concerns about the implications of older people’s changing living arrangements for public and health policies. Although countries vary in their approach to social welfare, in Mexico families remain the most prevalent source of support for older adults due to strong familialism, economic disadvantage, and limited access to social protection. Responding to the needs of aging populations requires an understanding of the dynamics of older adults’ living arrangements.

Compared to previous cross-sectional studies, our accelerated longitudinal approach coupled with latent class analysis gives a more complex picture of older people’s household dynamics in Mexico. Unlike previous studies in the region, our results do not show typical transitions between theoretical household structures (nuclear, extended, single-person), but typical household trajectories comprised of different mixtures of the said structures. It is important to note that we do not claim our data-driven exploratory approach is better, but complementary. We hope to have shown the usefulness of probing the possibilities of the survey data at hand, fully recognizing both its limitations and potential. With this ALD-LCA approach we can now look at how different health and wealth trajectories correlate with these typical histories of Mexican household structures (as per the MHAS), throwing new light on the role different living arrangements play in the well-being of older adults. This, however, remains pending work.

Understanding changes in living arrangements as people grow older should contribute to and inform policy development, specifically concerning health, access to health care, and caregiving. Knowing how health-related outcomes correlate with typical living arrangement trajectories offers policymakers a way to identify particularly vulnerable populations and to understand the implications of living alone and intergenerational support on mental health, and which aspects of family ties play an important role in shaping older people’s living conditions. Given that recently an increasing number of migrants to the United States have been returning to Mexico (Denier and Masferrer 2019; Gutiérrez Vázquez 2019), and that possibly this group includes older adults, research on living arrangements using different techniques may also inform policies targeting ‘at-risk’ individuals, such as these return migrants. However, more research is necessary in order to understand the gender dimensions of aging and to determine why certain individuals are more at risk of experiencing negative health and economic shocks. As living arrangement dynamics in Mexico and Latin America are in all likelihood strongly gendered, having a sense of the likely health-related outcomes of an older woman in a relatively small household may prove invaluable.

Understanding the relationship between living arrangement dynamics and the use of health care services at older ages may also provide policymakers with valuable information regarding health-related outcomes, all of which could help when considering the policies and services required to improve the well-being of older adults and when designing reasonable retirement plans ‒ a point that has been made in Mexico for quite some time now (Montes de Oca 1999). We believe these methodological tools have the potential to stimulate new research agendas aimed at strengthening social security networks for the greater good of low- and middle-income countries.

## Figures and Tables

**Table 1:** Number of observations by wave and age group

	Age (sd)									
Wave	52 (1.1)	55 (1.4)	59 (1.4)	63 (1.4)	67 (1.3)	71 (2.1)	75 (2.3)	81 (4.0)	86 (4.4)	*n*
2001	2,804	2,447	2,023	1,674	1,348	1,339	688	696	11	13,030
2003	0	2,598	2,288	1,891	1,584	1,283	1,305	640	667	12,256
2012	1,663	2,008	1,508	2,473	1,987	1,536	1,216	1,556	393	14,340
2015	0	1,460	1,803	1,344	2,345	1,921	1,407	1,077	1,468	12,825
Total	4,467	8,513	7,622	7,382	7,264	6,079	4,616	3,969	2,539	52,451

*Source*: Prepared by authors based on data from MHAS I-IV.

**Table 2:** Number of observations by age group and cohort in the MHAS-ALD

	Age (sd)									
	52 (1.1)	55 (1.4)	59 (1.4)	63 (1.4)	67 (1.3)	71 (2.1)	75 (2.3)	81 (4.0)	86 (4.4)	*n*
Age groups	[50–53]	[54–57]	[58–61]	[62–65]	[66–69]	[70–73]	[74–77]	[78–81]	[82–]	
Cohort										
12	1,663	1,460	–	–	–	–	–	–	–	3,123
11	–	2,008	1,803	–	–	–	–	–	–	3,811
10	–	–	1,508	1,344	–	–	–	–	–	2,852
9	2,804	2,598	–	2,473	2,345	–	–	–	–	10,220
8	–	2,447	2,288	–	1,987	1,921	–	–	–	8,643
7	–	–	2,023	1,891	–	1,536	1,407	–	–	6,857
6	–	–	–	1,674	1,584	–	1,216	1,077	–	5,551
5	–	–	–	–	1,348	1,283	–	1,556	1,468	5,655
4	–	–	–	–	–	1,339	1,305	–	393	3,037
3	–	–	–	–	–	–	688	640	–	1,328
2	–	–	–	–	–	–	–	696	667	1,363
1	–	–	–	–	–	–	–	–	11	11
Total	4,467	8,513	7,622	7,382	7,264	6,079	4,616	3,969	2,539	52,451

*Source*: Prepared by authors based on data from MHAS I-IV.

**Table 3:** Changes in household structure characteristics by time-period (index older adult mean age) in the accelerated longitudinal design

Time period	1	2	3	4	5	6	7	8	9
***Demographic variables***									
Index older adult average age	51.6	54.8	58.6	62.7	66.7	70.9	75.0	80.5	84.7
Index older adult sex (Proportion women)	.56	.57	.57	.54	.52	.53	.54	.54	.56
Household average size	4.4	4.2	4.0	3.8	3.6	3.5	3.4	3.3	3.2
***Household dichotomous demographic variables (1–Yes, 0–No). Percentages***									
Children living in the household	82.2	78.0	70.7	64.8	59.6	55.9	54.2	54.3	55.1
Children < 6 years old	3.0	1.6	1.1	0.7	0.4	0.3	0.1	0.2	0.1
Children ≥ 6 and ≤ 11 years old	12.4	6.8	3.6	2.0	1.3	0.9	0.6	0.3	0.1
Children ≥ 12 and ≤ 14 years old	16.6	10.3	5.4	2.9	1.5	0.9	0.6	0.6	0.2
Children ≥ 15 and ≤ 17 years old	25.5	18.1	10.7	5.0	2.7	1.3	1.0	0.6	0.4
Children ≥ 12 and ≤ 17 years old	35.1	24.1	14.1	7.1	3.7	1.9	1.5	1.1	0.5
Children ≥ 18 years old	69.5	70.1	65.7	62.0	58.0	54.9	53.4	53.7	54.8
Student children ≥ 18 years old	12.9	10.5	6.7	4.2	2.4	1.4	0.7	0.5	0.3
Working children (aged ≥ 13)	50.4	52.4	51.1	50.2	47.4	43.8	41.3	40.0	38.0
Working children ≥ 18 years old	48.2	50.9	50.3	49.7	47.1	43.6	41.2	39.9	37.9
Children’s partners	11.6	12.6	13.5	13.3	14.1	14.5	14.6	15.8	17.2
Other household members’ death	0.3	1.5	1.9	3.2	3.0	3.1	2.6	3.3	2.8
Index older adult’s partner or spouse	77.8	77.1	74.1	72.3	69.0	62.0	54.6	44.3	35.2
Nonrelatives	0.8	0.9	0.7	1.0	0.9	1.0	1.4	1.6	2.4
Other relatives	31.6	34.1	37.3	36.7	37.4	38.0	38.5	40.0	40.8
Index older adult’s parents and/or in-laws	6.3	5.6	4.7	3.4	1.8	1.2	0.6	0.5	0.6
Grandchildren and/or great grandchildren	20.8	24.3	28.4	29.5	31.7	32.9	33.7	34.4	34.3
Index older adult’s partners aged 40 or less	3.5	2.0	1.3	0.7	0.4	0.4	0.2	0.2	0.2
Index older adult partner’s death	0.7	1.3	1.8	3.2	4.9	5.9	7.0	10.9	7.7

*Source*: Prepared by authors based on data from MHAS I-IV.

**Table 4:** Determinants of the optimal number of living arrangement trajectory latent classes

(1)	(2)	(3)	(4)	(5)			(6)		
Class	−2xlog-L	BIC	Entropy	Relative class size (%)			Range of probabilities		
1	581,815	582,428	–	–	–	–	–	–	–
2	504,621	505,841	0.965	41	–	59	0.974	–	0.976
3	469,394	471,220	0.978	31	–	36	0.971	–	0.986
4	446,711	449,144	0.973	20	–	28	0.956	–	0.980
5	433,407	436,446	0.967	17	–	22	0.954	–	0.974

*Source*: Prepared by authors based on data from MHAS I-IV.

## Footnotes

4 According to the World Bank (2014), Mexico has some of the lowest rates of available healthcare resources in Latin America, particularly regarding hospital beds and physicians per 1,000 people (Payne 2015).

5 Also known as mixed longitudinal, cross-sequential, or cohort-sequential design (Baltes and Nesselroade 1979).

6 Note that cohorts 10, 11, and 12 belong to the 2012 sample refreshment and therefore are only seen twice at best.

7 The small number of respondents in the oldest age group at baseline (11) may come as a surprise, but keep in mind that the working sample removed all age inconsistencies.

8 Although there is no general formula for assessing the validity of a particular ALD, recovering information concerning the full longitudinal effect from different cohort segments depends on the number of subjects and cohorts, the degree of overlap between cohorts, and the frequency of observations (Moerbeek 2011).

9 Note that in single-cohort longitudinal designs, age and history are perfectly confounded. Time-dependent deflections may represent historical effects as well as age effects (Miyazaki and Raudenbush 2000).

10 When age–outcome trajectories do not vary by cohort, they may be said to converge (Miyazaki and Raudenbush 2000).

11 This multi-group analysis is an extension of the standard LCA used to compare the latent structure derived from a set of discrete item responses between multiple groups (Kankaraš, Moors, and Vermunt 2010).

12 We estimated three LCA models in parallel, holding all thresholds equal to obtain an LCA model with measurement invariance and equal transition probabilities.

13 It is important to note that even though we used the multigroup LCA to force a latent structure derived from different cohorts, this approach allows researchers to qualify in detail the differences observed across cohorts – which is beyond the scope of this article and is left for future research.

14 Due to nonconvergence on the global maximum of the likelihood, we show no results for more than 5 latent classes. Note that the eventual nonconvergence of these kinds of models is bound to happen, as the number of parameters to be estimated grows arithmetically with the number of classes. In this case, a 5-class model already estimates 183 parameters. In this way, every additional class adds to the computational burden, straining the data to the point of nonconvergence.

15 The estimated 3-class model, for example, roughly distributes Class 1 into Classes 2 and 3 in the 4-class model discussed above.

## Appendix B

**Table A-5:** Distribution of older adults per locality size by class

	Classes				
Locality size	1	2	3	4	Total
100,000–+	2,302	3,055	3,369	2,826	11,552
15,000–99,999	502	614	658	566	2,340
2,500–14,999	396	444	436	482	1,758
< 2,500	562	865	796	972	3,195
Total	3,762	4,978	5,259	4,846	18,845

*Source*: Prepared by authors based on data from MHAS I-IV.

**Table A-6:** Household structure by age. Class 1

Time-periods	1	2	3	4	5	6	7	8	9
Variables									
Age (Years)	51.6	54.8	58.6	62.7	66.7	71.0	75.2	80.8	86.1
Sex (Proportion women)	.65	.67	.67	.63	.60	.61	.60	.59	.62
Household size	6.4	6.3	6.3	6.2	6.0	6.0	6.0	5.6	5.2
Presence in the household of (1-Yes 2-No). Percentages									
Children	98.5	97.3	97.8	97.1	94.8	97.3	98.6	97.8	96.7
Children less than 6 years old	2.0	1.3	0.9	0.9	0.3	0.0	0.0	0.2	0.0
Children between 6 and 11 years old	8.1	4.8	2.2	1.0	0.4	0.5	0.8	0.4	0.2
Children between 12 and 14 years old	12.9	6.8	3.9	2.5	1.3	0.5	0.7	0.4	0.2
Children between 15 and 17 years old	22.2	15.1	9.0	4.8	2.2	1.4	0.9	0.9	0.2
Children between 12 and 17 years old	31.0	19.6	11.9	6.8	3.5	1.7	1.4	1.3	0.4
Children 18 years old and over	97.0	95.7	96.9	96.6	94.6	97.1	98.5	97.8	96.7
Children 18 years old and over (Students)	6.6	4.2	2.8	2.3	1.0	1.0	0.4	0.8	0.7
Children that work	80.0	79.7	81.0	81.7	78.4	80.0	77.8	75.2	72.2
Children 18 years old and over that work	78.8	78.6	80.4	81.3	78.3	79.9	77.8	75.2	72.2
Children’s partners	45.7	46.3	46.7	44.5	45.9	46.7	49.7	50.4	48.3
Death of a household member	0.3	1.7	2.2	3.6	3.7	4.0	3.2	4.3	4.4
Partner	75.8	73.1	68.7	65.7	59.0	51.4	41.5	33.6	26.5
Nonrelatives	1.0	1.1	0.8	1.3	0.9	1.2	2.1	1.8	2.7
Other relatives	89.1	92.3	96.9	94.7	93.7	93.5	97.7	96.6	89.8
Ascendants	4.3	5.2	4.2	2.6	1.3	1.0	0.6	0.8	1.8
Further descendants	81.0	85.4	91.9	90.6	90.2	89.9	94.6	92.5	83.1
Partner female under 41	1.5	0.4	0.1	0.1	0.1	0.2	0.1	0.0	0.0
Partner’s death	1.6	1.6	1.6	4.5	5.6	6.2	7.8	7.1	6.4

*Source*: Prepared by authors based on data from MHAS I-IV.

**Table A-7:** Household structure by age. Class 2

Time-periods	1	2	3	4	5	6	7	8	9
Variables									
Age (Years)	51.6	54.7	58.6	62.8	66.6	70.8	75.0	80.4	85.5
Sex (Proportion women)	0.0	0.1	0.0	0.0	0.0	0.0	0.0	0.0	0.0
Household size	4.6	4.5	4.3	3.9	3.8	3.6	3.4	3.5	3.5
Presence in the household of (1-Yes 2-No). Percentages									
Children	96.3	95.7	90.4	80.8	73.4	67.1	65.4	69.2	72.9
Children less than 6 years old	7.6	4.2	3.0	1.5	1.0	0.9	0.3	0.4	0.2
Children between 6 and 11 years old	23.1	15.4	9.4	5.1	3.2	2.8	1.3	1.1	0.2
Children between 12 and 14 years old	25.6	19.5	13.1	6.7	3.9	2.7	1.8	1.8	0.9
Children between 15 and 17 years old	36.6	28.8	20.6	11.6	6.6	3.5	2.8	1.4	1.1
Children between 12 and 17 years old	50.6	39.8	28.3	15.8	9.1	5.3	4.2	2.9	1.7
Children 18 years old and over	75.5	81.2	79.0	74.1	69.2	64.0	62.9	66.9	71.8
Children 18 years old and over (Students)	18.1	16.4	11.7	8.4	5.5	3.2	1.6	0.9	0.6
Children that work	51.5	57.1	59.4	58.0	55.8	50.0	48.4	50.6	50.0
Children 18 years old and over that work	48.7	54.7	57.7	56.7	55.0	49.6	48.1	50.3	49.8
Children’s partners	3.8	5.5	8.4	8.5	9.7	9.4	7.0	10.4	14.4
Death of a household member	0.3	0.9	1.6	2.8	2.3	2.8	2.6	3.1	3.2
Partner	95.6	95.2	94.9	92.4	89.6	86.7	82.1	71.7	57.6
Nonrelatives	0.6	0.5	0.4	0.7	0.7	0.7	0.8	0.8	1.1
Other relatives	12.7	16.6	22.5	22.9	25.6	25.9	23.4	27.0	31.7
Ascendants	3.3	3.5	2.8	2.5	1.7	1.3	1.0	0.8	0.7
Further descendants	6.3	10.1	15.3	17.2	20.4	21.9	20.0	23.6	27.8
Partner female under 41	9.6	5.9	4.3	2.3	1.3	1.2	0.6	0.6	0.4
Partner’s death	0.0	0.4	0.5	1.7	3.0	3.8	5.7	10.9	11.2

*Source*: Prepared by authors based on data from MHAS I-IV.

**Table A-8:** Household structure by age. Class 3

Time-periods	1	2	3	4	5	6	7	8	9
Variables									
Age (Years)	51.5	54.7	58.5	62.7	66.6	70.9	75.0	80.7	85.6
Sex (Proportion women)	1	1	1	1	1	1	1	1	1
Household Size	4.2	4.0	3.7	3.4	3.2	3.1	2.9	2.9	3.0
Presence in the Household of (1-Yes 2-No). Percentages									
Children	0.5	0.2	0.2	0.2	0.1	0.1	0.1	0.0	0.0
Children less than 6 years old	9.9	3.7	1.8	0.8	0.5	0.2	0.2	0.0	0.0
Children between 6 and 11 years old	16.2	9.1	3.1	1.3	0.3	0.2	0.0	0.2	0.0
Children between 12 and 14 years old	29.1	19.1	9.8	2.4	0.8	0.2	0.2	0.3	0.2
Children between 15 and 17 years old	37.6	24.2	11.8	3.5	1.0	0.4	0.2	0.5	0.2
Children between 12 and 17 years old	83.6	87.5	83.5	75.5	65.1	63.6	64.4	70.3	77.8
Children 18 years old and over	19.1	15.3	9.8	4.4	1.7	1.1	0.7	0.4	0.2
Children 18 years old and over (Students)	58.7	64.4	63.2	61.0	53.6	50.3	48.6	49.5	48.9
Children that work	55.8	62.6	62.5	60.7	53.5	50.3	48.5	49.3	48.9
Children 18 years old and over that work	4.7	7.2	7.3	8.1	8.8	9.4	9.0	10.5	15.3
Children’s partners	0.2	1.4	1.8	3.2	3.1	3.1	2.7	4.0	3.0
Death of a Household member	74.3	73.8	68.2	63.3	59.1	49.8	42.4	26.9	16.6
Partner	0.7	0.6	0.6	0.6	0.9	0.6	1.2	1.4	1.5
Nonrelatives	16.5	21.4	22.4	23.7	26.1	27.2	26.2	28.5	31.9
Other relatives	4.8	4.2	3.4	2.2	1.4	1.1	0.3	0.2	0.2
Ascendants	7.9	12.6	15.3	18.1	21.6	23.4	22.6	24.6	26.6
Further descendants	0.0	0.0	0.0	0.0	0.0	0.0	0.0	0.0	0.0
Partner female under 41	1.1	1.8	2.6	5.1	6.8	8.2	9.5	14.8	7.2
Partner’s death	0.5	0.2	0.2	0.2	0.1	0.1	0.1	0.0	0.0

*Source*: Prepared by authors based on data from MHAS I-IV.

**Table A-9:** Household structure by age. Class 4

Time-periods	1	2	3	4	5	6	7	8	9
Variables									
Age (Years)	51.7	54.9	58.7	62.6	66.7	71.1	75.1	80.9	85.7
Sex (Proportion women)	.58	.58	.58	.56	.54	.53	.54	.54	.56
Household Size	2.7	2.3	2.2	2.1	2.0	2.0	2.0	1.9	1.9
Presence in the Household of (1-Yes 2-No). Percentages									
Children	25.5	8.3	3.1	1.6	1.6	0.7	0.2	0.2	0.1
Children less than 6 years old	1.3	0.5	0.3	0.2	0.1	0.0	0.0	0.0	0.1
Children between 6 and 11 years old	4.9	1.6	0.5	0.6	0.2	0.1	0.1	0.0	0.0
Children between 12 and 14 years old	7.2	2.5	0.7	0.5	0.1	0.0	0.2	0.1	0.0
Children between 15 and 17 years old	6.1	4.0	1.9	0.4	0.1	0.1	0.0	0.1	0.1
Children between 12 and 17 years old	11.4	5.8	2.3	0.8	0.3	0.1	0.2	0.2	0.1
Children 18 years old and over	11.3	1.3	0.3	0.4	1.1	0.6	0.0	0.0	0.0
Children 18 years old and over (Students)	0.5	0.0	0.0	0.0	0.0	0.0	0.0	0.0	0.0
Children that work	7.1	0.2	0.2	0.0	0.6	0.1	0.0	0.0	0.0
Children 18 years old and over that work	6.3	0.1	0.1	0.0	0.6	0.1	0.0	0.0	0.0
Children’s partners	3.3	0.1	0.0	0.1	0.1	0.0	0.0	0.0	0.0
Death of a Household member	0.5	2.5	2.0	3.5	3.4	2.9	2.0	1.9	1.4
Partner	59.0	60.3	62.3	63.9	62.4	56.4	51.3	45.6	40.0
Nonrelatives	1.1	1.5	1.1	1.7	1.5	1.6	1.7	2.2	3.8
Other relatives	32.5	25.7	24.5	22.4	20.6	19.1	19.0	19.3	20.9
Ascendants	15.2	11.3	9.3	6.8	2.7	1.5	0.5	0.2	0.0
Further descendants	8.6	6.9	8.0	8.7	10.6	10.3	10.4	9.9	12.1
Partner female under 41	2.2	1.1	0.7	0.3	0.1	0.0	0.0	0.2	0.2
Partner’s death	0.0	1.5	2.2	1.6	4.2	5.4	5.0	10.1	6.6

*Source*: Prepared by authors based on data from MHAS I-IV.

## References

[R1] Aguila E, Diaz C, Fu MM, Kapteyn A, and Pierson A (2011). Living longer in Mexico: Income security and health. San Cristóbal: RAND Corporation, Centro Fox (AARP 2011) https://www.rand.org/pubs/monographs/MG1179.html. 10.7249/rhq-01-04-01 PMC4945250 28083208

[R2] Angel JL, Vega W, and López-Ortega M (2017). Aging in Mexico: Population trends and emerging issues. The Gerontologist 57(2): 153‒162. 27927730 10.1093/geront/gnw136 PMC5881744

[R3] Baltes PB and Nesselroade JR (1979). Longitudinal research in the study of behavior and development. New York: Academic Press.

[R4] Becker GS (1991). A treatise on the family. Cambridge: Harvard University Press.

[R5] Bell RQ (1953). Convergence: An accelerated longitudinal approach. Child Development 24(2): 145‒152. doi:10.2307/1126345. 13141335

[R6] Bernabe-Ortiz A, Diez-Canseco F, Vásquez A, and Miranda JJ (2016). Disability, caregiver’s dependency and patterns of access to rehabilitation care: results from a national representative study in Peru. Disability and Rehabilitation 38(6): 582‒588. doi:10.3109/09638288.2015.1051246. 26017542

[R7] Bitran R (2014). Universal health coverage and the challenge of informal employment: Lessons from developing countries. Washington, D.C.: World Bank Group (Health, Nutrition, and Population (HNP) discussion paper). http://documents.worldbank.org/curated/en/698041468180275003/Universal-health-coverage-and-the-challenge-of-informal-employment-lessons-from-developing-countries.

[R8] Bobadilla JL, Frenk J, Lozano R, Frejka T, and Stern C (1993). The epidemiologic transition and health priorities In: Jamison DR, Mosley WH, Measham AR, and Banadilla JL (eds.). Disease control priorities in developing countries. Oxford: Oxford University Press: 51–63.

[R9] Brown JW, Liang J, Krause N, Akiyama H, Sugisawa H, and Fukaya T (2002). Transitions in living arrangements among elders in Japan: does health make a difference? The Journals of Gerontology Series B: Psychological Sciences and Social Sciences 57(4): S209‒S220. doi:10.1093/geronb/57.4.S209. 12084791

[R10] Ceballos Mina OE (2017). Personas mayores en México: perfiles de consumo y otros efectos económicos en sus hogares In: Montes de Oca Zavala V and Nava Bolaños I (eds.). Población y envejecimiento: Pasado, presente y futuro en la investigación sociodemográfica. Mexico City: UNAM: 201‒230.

[R11] Celeux G and Soromenho G (1996). An entropy criterion for assessing the number of clusters in a mixture model. Journal of Classification 13(2): 195‒212. doi:10.1007/BF01246098.

[R12] Chant SH (2007). Gender, generation and poverty: Exploring the feminisation of poverty in Africa, Asia and Latin America. Cheltenham: Edward Elgar Publishing.

[R13] Cortés F (2000). La distribución del ingreso en México en épocas de estabilización y reforma económica. Mexico City: Porrúa-CIESAS.

[R14] Cortés F and Rubalcava RM (1991). Autoexplotación forzada y equidad por empobrecimiento: La distribución del ingreso familiar en México (1977‒1984). Mexico City: El Colegio de México. doi:10.2307/j.ctv6mtc8v.

[R15] Cortés F and Vargas-Chanes D (2016). Dos décadas de marginación en México: Un enfoque longitudinal. Mexico City: Universidad Nacional Autónoma de México.

[R16] De Vos S (1990). Extended family living among older people in six Latin American countries. Journal of Gerontology 45(3): S87‒S94. doi:10.1093/geronj/45.3.S87. 2335736

[R17] DeGraff DS, Wong R, and Orozco-Rocha K (2018). Dynamics of economic security among the aging in Mexico: 2001–2012. Population Research and Policy Review 37(1): 59‒90. doi:10.1007/s11113-017-9449-x. 30250353 PMC6150599

[R18] Denier N and Masferrer C (2019). Returning to a New Mexican labor market? Regional variation in the economic incorporation of return migrants from the US to Mexico. Population Research and Policy Review. online first: 1‒25. doi:10.1007/s11113-019-09547-w.

[R19] Dostie B and Léger PT (2005). The living arrangement dynamics of sick, elderly individuals. Journal of Human Resources 40(4): 989‒1014. doi:10.3368/jhr.XL.4.989.

[R20] Du RY and Kamakura WA (2006). Household life cycles and lifestyles in the United States. Journal of Marketing Research 43(1): 121‒132. doi:10.1509/jmkr.43.1.121.

[R21] Duncan SC, Duncan TE, and Hops H (1996). Analysis of longitudinal data within accelerated longitudinal designs. Psychological Methods 1(3): 236‒248. doi:10.1037//1082-989X.1.3.236.

[R22] Duncan TE, Duncan SC, and Strycker LA (2013). An introduction to latent variable growth curve modeling: Concepts, issues, and application. New York: Routledge Academic. doi:10.4324/9780203879962.

[R23] Economic Commission for Latin America and the Caribbean (ECLAC) (2019). Social panorama of Latin America, 2018. Santiago (LC/PUB.2019/3-P).

[R24] Farrington DP (1991). Longitudinal research strategies: Advantages, problems, and prospects. Journal of the American Academy of Child and Adolescent Psychiatry 30(3): 369‒374. doi:10.1097/00004583-199105000-00003. 2055872

[R25] Garay Villegas S, Montes de Oca Zavala V, and Guillén J (2014). Social support and social networks among the elderly in Mexico. Journal of Population Ageing 7(2): 143‒159. doi:10.1007/s12062-014-9099-2.

[R26] Garay Villegas S and Montes de Oca Zavala V (2011). La vejez en Mexico: Una mirada general sobre la situacion socioeconomica y familiar de los hombres y mujeres adultos mayores. Perspectivas Sociales 13(1): 143‒165.

[R27] González de la Rocha M (1994). The resources of poverty: Women and survival in a Mexican city. Oxford: Blackwell.

[R28] González KD (2015). Envejecimiento demográfico en México: análisis comparativo entre las entidades federativas In: Consejo Nacional de Población (eds.). La situación demográfica de México. Mexico City: Consejo Nacional de Población: 113‒129.

[R29] Gruijters RJ (2017). Family care-giving and living arrangements of functionally impaired elders in rural China. Ageing and Society 37(3): 633‒655. doi:10.1017/S0144686X15001397.

[R30] Guo S and Fraser MW (2015). Propensity score analysis. Thousand Oaks: Sage.

[R31] Gutiérrez Vázquez EY (2019). The 2000‒2010 changes in labor market incorporation of return Mexican migrants. Revista Latinoamericana de Población 13(24): 135‒162. doi:10.31406/relap2019.v13.i1.n24.6.

[R32] Hagenaars JA and McCutcheon AL (2002). Applied latent class analysis. Cambridge: Cambridge University Press. doi:10.1017/CBO9780511499531.

[R33] Ham Chande R (2010). Envejecimiento demográfico In: García B and Ordorica M(eds.). Población. Mexico City: El Colegio de México: 53‒78.

[R34] Ham Chande R (2014). El envejecimiento en México: el siguiente reto de la transición demográfica. Tijuana: El Colegio de la Frontera Norte.

[R35] Kanaiaupuni SM (2000). Leaving parents behind: Migration and elderly living arrangements in Mexico. Madison: Center for Demography and Ecology, University of Wisconsin-Madison (Working paper 1999‒16).

[R36] Kankaraš M, Moors G, and Vermunt JK (2010). Testing for measurement invariance with latent class analysis In: Davidov E, Schmidt P, and Billiet J (eds.). Cross-cultural analysis: Methods and applications. New York: Routledge Taylor and Francis Group: 359‒384.

[R37] Kawachi I, Subramanian SV, and Kim D (2008). Social capital and health In: Kawachi I, Subramanian SV, and Kim D (eds.). Social capital and health. New York: Springer: 1‒26. doi:10.1007/978-0-387-71311-3.

[R38] Keilman N and Prinz C (1995). Modelling the dynamics of living arrangements In: Gonnot J-P, Keilman N, and Prinz C (eds.). Social security, household, and family dynamics in ageing societies: European studies of population, Vol 1. Dordrecht: Springer: 21‒45. doi:10.1007/978-94-015-8441-8_2.

[R39] Kuznets S (1978). Size and age structure of family households: Exploratory comparisons. Population and Development Review 4(2): 187‒223. doi:10.2307/1972278.

[R40] Lo Y, Mendell NR, and Rubin DB (2001). Testing the number of components in a normal mixture. Biometrika 88(3): 767‒778. doi:10.1093/biomet/88.3.767.

[R41] Lloyd-Sherlock P, Mayston R, Acosta A, Gallardo S, Guerra M, Sosa AL, and Prince M (2018). Allocating family responsibilities for dependent older people in Mexico and Peru. The Journal of Development Studies 54(4): 682‒701. doi:10.1080/00220388.2017.1308489.

[R42] Manfredini M and Breschi M (2013). Living arrangements and the elderly: An analysis of old-age mortality by household structure in Casalguidi, 1819–1859. Demography 50(5): 1593‒1613. doi:10.1007/s13524-013-0218-0. 23686383

[R43] Martikainen P, Nihtilä E, and Moustgaard H (2008). The effects of socioeconomic status and health on transitions in living arrangements and mortality: A longitudinal analysis of elderly Finnish men and women from 1997 to 2002. *The Journals of Gerontology Series B*: Psychological Sciences and Social Sciences 63(2): S99‒S109. doi:10.1093/geronb/63.2.S99. 18441275

[R44] Miyazaki Y and Raudenbush SW (2000). Tests for linkage of multiple cohorts in an accelerated longitudinal design. Psychological Methods 5(1): 44‒63. doi:10.1037//1082-989X.5.1.44. 10937322

[R45] Moerbeek M (2011). The effects of the number of cohorts, degree of overlap among cohorts, and frequency of observation on power in accelerated longitudinal designs. Methodology 7(1): 11‒24. doi:10.1027/1614-2241/a000019.

[R46] Montes de Oca V (1999). Relaciones familiares y redes sociales Envejecimiento demográfico en México: Retos y perspectivas. Por una sociedad para todas las edades. Mexico City: Consejo Nacional de Población: 290‒326.

[R47] Montes de Oca V, Garay S, Rico B, and García SJ (2014). Living arrangements and aging in Mexico: Changes in households, poverty and regions, 1992‒2009. International Journal of Social Science Studies 2(4): 61‒74. doi:10.11114/ijsss.v2i4.453.

[R48] Montes de Oca V and Hebrero M (2006). Eventos cruciales y ciclos familiares avanzados: El efecto del envejecimiento en los hogares de México. Papeles de población 12(50): 97‒116. PMC5790122 29391856

[R49] Montes de Oca V and Hebrero M (2008). Family dynamics, aging, and functional impairment in Mexico. *Revista Kairos*: Gerontologia 11(1): 143‒166. PMC4548963 26317060

[R50] Montes de Oca Zavala V and Nava Bolaños I (eds.) (2017). Población y envejecimiento: pasado, presente y futuro en la investigación sociodemográfica. Mexico City: Universidad Nacional Autónoma de México.

[R51] Muennig P, Jiao B, and Singer E (2018). Living with parents or grandparents increases social capital and survival: 2014 General Social Survey-National Death Index. SSM-Population Health 4: 71‒75. doi:10.1016/j.ssmph.2017.11.001. 29349275 PMC5769098

[R52] Murphy M (1991). Household modelling and forecasting: Dynamic approaches with use of linked Census data. Environment and Planning A 23(6): 885‒902. doi:10.1068/a230885. 12284182

[R53] Nava Bolaños I and Acosta L (2018). Conexiones demográficas. Isalia Nava Bolaños y Laura Acosta en diálogo con Roberto Ham Chande. Revista Latinoamericana de Población 12(22): 106‒109. doi:10.31406/n22a7.

[R54] Nava Bolaños I, Ham Chande R, and Ramírez López BP (2016). Seguridad económica y vejez en México. Revista Latinoamericana de Población 10(19): 169‒190. doi:10.31406/relap2016.v10.i2.n19.8.

[R55] Oberski D (2016). Mixture models: Latent profile and latent class analysis In: Robertson J and Kaptein M (eds.). Modern statistical methods for HCI. Cham:Springer: 275‒287. doi:10.1007/978-3-319-26633-6_12.

[R56] Parker SW, Saenz J, and Wong R (2018). Health insurance and the aging: Evidence from the Seguro Popular program in Mexico. Demography 55(1): 361‒386. doi:10.1007/s13524-017-0645-4. 29357097 PMC5829015

[R57] Partida-Bush V (2006). Situación demográfica nacional y estatal. Mexico City:Consejo Nacional de Población.

[R58] Payne CF (2015). Aging in the Americas: Disability-free life expectancy among adults aged 65 and older in the United States, Costa Rica, Mexico, and Puerto Rico. The Journals of Gerontology: Series B 73(2): 337‒348. doi:10.1093/geronb/gbv076. PMC6283317 26347520

[R59] Pérez Amador J and Brenes G (2006). Una transición en edades avanzadas: Cambios en los arreglos residenciales de adultos mayores en siete ciudades latinoamericanas. Estudios Demográficos y Urbanos 21(3): 625‒661. doi:10.24201/edu.v21i3.1243.

[R60] Ramírez López BP, Nava Bolaños I, and Badillo González G (2018). Las raíces de la desigualdad y de la exclusión previsional en México: Propuesta para su rediseño In: Rodríguez I and Vommaro P (eds.). Desigualdades, exclusión y crisis de sustentabilidad en los sistemas previsionaels de América Latina y el Caribe. Buenos Aires: CLACSO-CLATE: 143‒171. doi:10.2307/j.ctvfp62vr.9.

[R61] Redondo N, Garay S, and Montes de Oca V (2015). Modalidades de allegamiento residencial en la población adulta mayor argentina y mexicana: Determinantes socioeconómicos y diferencias regionales. Estudios Demográficos y Urbanos 30(3): 597‒649. doi:10.24201/edu.v30i3.1495.

[R62] Regules García R (2018). Notas sobre el futuro demográfico de México y el binomio población y desarrollo. Mexico City: Programa Universitario de Estudios del Desarrollo, UNAM.

[R63] Salgado-de Snyder VN and Wong R (2007). Género y pobreza: determinantes de la salud en la vejez. Salud pública de México 49(S4): 515‒521. doi:10.1590/S0036-36342007001000011. 17724525

[R64] Sánchez L and Escoto A (2017). Arreglos residenciales multigeneracionales y pobreza en México. Coyuntura Demográfica 12: 71‒77.

[R65] Schwarz G (1978). Estimating the dimension of a model. The Annals of Statistics 6(2):461‒464. doi:10.1214/aos/1176344136.

[R66] Singh N (2002). Population and poverty. New Delhi: Mittal Publications.

[R67] Teerawichitchainan B, Pothisiri W, and Long GT (2015). How do living arrangements and intergenerational support matter for psychological health of elderly parents? Evidence from Myanmar, Vietnam, and Thailand. Social Science and Medicine 136–137: 106‒116. doi:10.1016/j.socscimed.2015.05.019. 25993521

[R68] The World Bank (2014). World development indicators. International Bank for Reconstruction and Development/The World Bank.

[R69] Thornton A, Chang M-C, and Sun T-H (1984). Social and economic change, intergenerational relationships, and family formation in Taiwan. Demography 21(4): 475‒499. doi:10.2307/2060911. 6519319

[R70] Tohme RA, Yount KM, Yassine S, Shideed O, and Sibai AM (2011). Socioeconomic resources and living arrangements of older adults in Lebanon: Who chooses to live alone? Ageing and Society 31(1): 1‒17. doi:10.1017/S0144686X10000590.

[R71] Ullmann H, Maldonado Valera C, and Nieves Rico M (2014). La evolución de las estructuras familiares en América Latina, 1990‒2010: Los retos de la pobreza, la vulnerabilidad y el cuidado (Vol. 193). Santiago: Naciones Unidas/Comisión Económica para América Latina y el Caribe (CEPAL).

[R72] United Nations Department of Economic and Social Affairs/Population Division (2005). Living arrangements of older persons around the world (Vol. 240). New York: United Nations Publications.

[R73] Varley A and Blasco M (2003). Older women’s living arrangements and family relationships in urban Mexico. Women’s Studies International Forum 26(6): 525‒539. doi:10.1016/j.wsif.2003.09.007.

[R74] Wang J and Wang X (2012). Structural equation modeling: Applications using Mplus. Hoboken: John Wiley and Sons. doi:10.1002/9781118356258.

[R75] Wedel M and Kamakura WA (2000). Market segmentation conceptual and methodological issues. Boston: Kluwer Academic Publishing.

[R76] Wong R, Michaels-Obregon A, and Palloni A (2015). Cohort profile: The Mexican health and aging study (MHAS). International Journal of Epidemiology 46(2): e2‒e2. doi:10.1093/ije/dyu263. PMC5837398 25626437

[R77] Ybáñez Zepeda E, Vargas Valle ED, and Torres Martín AL (2005). Factores asociados a la corresidencia de los adultos mayores de 50 anos por condicion rural-urbana. Papeles de Población 11(45): 29‒48. PMC5783557 29375251

